# A greater modified Mediterranean diet score is associated with lower insomnia score among adolescent girls: a cross-sectional study

**DOI:** 10.1186/s40795-022-00553-4

**Published:** 2022-06-29

**Authors:** Zahra Yaghtin, Sara Beigrezaei, Emad Yuzbashian, Majid Ghayour-Mobarhan, Sayyed Saeid Khayyatzadeh

**Affiliations:** 1 Department of Nutrition, School of Public Health, Shahid Sadoughi University of Medical Sciences, Yazd, Iran; 2 Nutrition and Food Security Research Center, Shahid Sadoughi University of Medical Sciences, Yazd, Iran; 3 Department of Agricultural, Food and Nutritional Science, University of Alberta, Edmonton, Alberta Canada; 4 Metabolic Syndrome Research Center, Mashhad University of Medical Sciences, Mashhad, Iran

**Keywords:** Modified Mediterranean diet, Insomnia, Sleep, Adolescent

## Abstract

**Background:**

Previous studies has shown that a low quality diet is related to sleep disorders. A Mediterranean diet is considered to be a high quality diet and has been shown to have beneficial effects on overall health. Thus, the aim of our study was to investigate the association between adherence to Mediterranean dietary pattern and insomnia score among adolescent girls.

**Methods:**

The data for 733 adolescent girls between 12–18 years old was assessed in this cross-sectional study. A 147 item-food frequency questionnaire was used to assess dietary intake. A modified model of Mediterranean diet score was calculated that ranged from 0–9 points. A validated version of Insomnia Severity Index questionnaire was used to assess insomnia. To explore the association between modified Mediterranean (mMED) diet score and insomnia, linear regression was conducted in crude and adjusted models (energy intake adjustmet in Model I, further adjustments were performed for physical activity, father’s and mother’s education in Model II and full adjusted model adjusted for age, body mass index percentiles, and abdominal obesity).

**Results:**

A significant inverse association between mMED diet score and insomnia score was observed using a crude model (β = -0.091, 95% confidence interval (CI): -0.392 to -0.046); *P*-value = 0.013) and also after adjustment for confounding factors in Model I (β = -0.098, CI: -0.423 to -0.045; *P* = 0.015), Model II (β = -0.092, CI: -0.410 to -0.029; *P*-value = 0.024), Model III (β = -0.082, CI: -0.385 to -0.006); *P* = 0.044).

**Conclusion:**

There was an inverse relationship between adherence to the mMED diet score and insomnia level among Iranian adolescent girls. Prospective studies are needed to confirm these results and clarify whether a causal relationship exists.

What is known? Adolescent girls are more likely to experience sleep problems such as longer sleep onset latency and insomnia than boys. Diet is believed to play a significant impact on sleeping health.

What is new? There are no data about the relationship between modified Mediterranean diet and insomnia especially in adolescent.

What is significant for clinical practice? Greater adherence to the mediterranean dietary pattern is less likely related to insomnia.

## Introduction

Sleep disorders have been shown to increase the risk of psychiatric illnesses and suicidal tendencies among adolescents [1]. Insomnia is one of the most common sleep disorders that have negative effects on overall health. It characterized by having persistent difficulty with sleep initiation, sleep maintenance or waking up early minimally three times a week, last for at least three months [2]. Higher risk of chronic headaches, irritable bowel syndrome, cardiovascular disease, cancer, Alzheimer’s diseases, and mental illnesses such as depression and anxiety followed by sleep disturbances [3–8].

Adolescence is a vulnerable stage of life in which individuals face physical and psychological maturity [9, 10]. Adolescent girls are more likely to experience sleep problems such as longer sleep onset latency and insomnia than boys [11]. Biological processes, electronic media, school time scheduling, and diet might contribute to sleep problems in adolescents [12]. Findings from previous large epidemiological studies indicated poor quality diet characterized by lower consumption of vegetables, fruits, and fish and higher consumption of processed foods and free-sugar-rich foods is associated with higher sleep problems [13–15]. A Mediterranean diet, which has been primarily used in Mediterranean regions, is considered to be a high quality dietary pattern due to high contents of plant-based foods, moderate contents of seafoods, eggs, poultry, dairy products, alcohol, and low contents of red meat [16]. A limited number of studies have shown a relationship between adherence to the Mediterranean diet and sleep especially among adolescent [17]. Adelantado-Renau et al. [18] have shown that sleep quality can be a significant mediator of the link between adherence to the Mediterranean diet and academic performance in adolescents. A cross-sectional study on adolescents has revealed that adherence to the Mediterranean diet was directly associated with sleep habits including sleep duration [19].

Given the limited available data about the potential association between the Mediterranean diet and insomnia in adolescents; we decided to examine the association between adherence to the mMED diet score and insomnia among a sample of adolescent girls in Iran.

## Method and material

### Study population and design

A total of 1026 adolescent girls aged 12–18 years were recruited by random cluster sampling method from several schools of various regions of Khorasan Razavi province, located in northern east Iran [20]. The girls with any cardiovascular disorders, renal or hepatic failure, cancer, malabsorption, thyroid, adrenal or parathyroid diseases, eating disorders, metabolic bone disease, and autoimmune diseases were excluded. In addition, we excluded subjects who were taking anti-inflammatory, antidiabetic or antiobesity drugs, antidepressants, vitamin D or calcium supplement use, and hormone therapy within the last six months (*n* = 38). In order to minimize the influence of under- and over reporting of energy intakes, subjects were omitted, if they reported the total energy intake out of the range of 800 to 4200 kcal/day (*n* = 255) [21]. Therefore, data from 733 participants were included in the final statistical analysis. All participants and their parents were asked to provide written consent. The survey was done based on the guidelines of the Helsinki Declaration. The Ethics Committee of Mashhad University of Medical Sciences (Code: 931,188) approved all procedures involving human patients, and the Department of Nutrition, Faculty of Medicine of this University, supported the study.

### Demographic and anthropometric assessment

Experienced interviewers collected demographic data using a standard questionaire. Physical activity levels was obtained by using the validated Modifiable Activity Questionnaire (MAQ) [22] and based on metabolic equivalent task (MET)-hour/day. Standard protocols were used to obtain the anthropometric variables, including weight, height and waist circumference (WC). Body Mass Index (BMI) was calculated by dividing weight (kilogram) by the squares of height (meters^2^). Abdominal obesity was defined as WC higher than or equal to the gender- and age-specific 90th percentile and WC to height ratio ≥ 0.5 [23].

### Dietary intake assessment

To obtain the dietary intake of participants, a food frequency questionnaire (FFQ) containing 147 food items was used. The validity and reliability of this has been previously reported [24]. The questionnaire was completed by face-to-face interview. The reported portion size in FFQ as converted to grams using household measures and the grams of food items were entered to the Nutritionist 4 software to estimate energy and nutrients intake. The food group intakes were calculated per 1000 Kcalorie to normalize the effect of energy intake.

### Adherence to modified Mediterranean (mMED) diet

The mMED diet score was calculated using previously published methods [25, 26]. Nine food groups including fruits, vegetables, legumes, nuts, whole grains, seafood, red meat, dairy products, and the ratio of monounsaturated fatty acid (MUFA) to saturated fatty acid (SFA) were used to compute this score. A score of 0 or 1 was assigned to each food item. We assigned a value of 0 to individual intakes below the median and and 1 to those above the median for whole grains, vegetables, fruits, legumes, nuts, seafood, and MUFA to SFA ratio. In addition, a score of 1 was considered for adolescents who consumed below the median and a 0 value for those with intake above the median level for red meat and dairy products. The mMED diet score ranged from 0 to 9.

### Assessment of insomnia

To assess insomnia, a validated Iranian version of the Insomnia Severity Index (ISI) questionnaire was used [27, 28]. The ISI questionnaire includes seven questions. The questionnaire’s score range is between 0 and 4 including four categories: 0 (None), 1 (Mild), 2 (Moderate), 3 (Severe), and 4 (Very Severe). Total score of ISI ranges between 0 and 28 points. If the ISI score was > 7, insomnia was difiend.

### Statistical methods

The participants were divided into three categories based on tertiles of their mMED diet scores. To compare the demographic characteristics of the study population, one-way-ANOVA and chi-squared analyses were applied across tertiles of mMED diet score for continuous variables including age (year), physical activity (MET-hour/day), insomnia (score) and categorical variables such as Mensturation status (yes/no), abdominal obesity (yes/no), BMI percentiles (3/ 5–85/ ≥ 85), and parents education (Illiterate/ Diploma/ Academic education), respectively. Energy-adjusted dietary intakes of study participants were compared by one-way ANOVA across tertiles of mMED diet score. Univariable and multivariable linear regressions were conducted in crude and adjusted models to examine the association between mMED diet score and insomnia level. In the adjusted models, this association was adjusted for energy intake in Model I. Additionally, further adjustments were made for mensturation status, physical activity, father’s and mother’s education in Model II. Final model were defined as further adjusting for age, BMI percentiles, and abdominal obesity. All statistical analyses were performed using the SPSS 22.0 (SPSS Corp, version 22, Chicago, IL, USA). *P*-values less than 0.05 were considered as statistically significant.

## Results

General characteristics of the study participants across tertile categories of mMED diet score are shown in Table 1. There were no significant differences for age, physical activity, mensturation status, and BMI percentiles between tertiles of the mMED diet score (*P* > 0.05). Significant differences were seen among tertiles of the mMED diet score for Father’s education (*P* = 0.025), Mother’s education (*P* = 0.006) and abdominal obesity (*P* = 0.012). Energy adjusted dietray intakes of study participants are provided in Table 2. The participants with a greater adherence to the mMED diet score had significant higher intakes of fruits (*P* < 0.001), vegetables (*P* < 0.001), legumes (*P* < 0.001), nuts (*P* = 0.012) and fish (*P* = 0.016) and lower consumption of total dariy (*P* < 0.001) and red meat (*P* = 0.003). In addition, the intake of total energy intake (*P* < 0.001), carbohydrate (*P* < 0.001), dietary fiber (*P* < 0.001), potassium (*P* = 0.012), beta-carotene (*P* < 0.001), vitamin C (*P* < 0.001), iron (*P* < 0.001) and folate (*P* = 0.006) was significantly higher among individuals who were in the third tertile of mMED diet score compared to those in the first tertile. However, The subjects who were categorized in the lowest tertile of mMED diet score consumed higher amounts of total fat (*P* = 0.004), cholesterol (*P* < 0.001), SFA (*P* < 0.001), MUFA (*P* < 0.001), PUFA (*P* < 0.001), MUFA/PUFA, sodium (*P* < 0.001) and calcium (*P* < 0.001) compared to those in the highest tertile.

**Table 1:** General characteristics of study participants by tertiles of mMED diet score

		Tertiles of mMED diet score (ranged from 0–9)			*P-value*^a^
		T1	T2	T3	
Age (y)		14.59 ± 1.5	14.48 ± 1.5	14.39 ± 1.5	0.312
Physical activity (MET. h/week)		45.34 ± 3.1	45.19 ± 3.1	45.59 ± 4.0	0.522
Abdominal obesity (%)		6.2	12.0	13.2	0.012
Mesturation (%)	Yes	51.2	21.9	26.9	0.302
	No	41.8	28.4	29.9	
BMI percentile (%)	3	3.2	1.2	1.5	0.220
	5–85	79.5	76.2	75.8	
	≥ 85	17.3	22.6	22.7	
Father’s education (%)	Illiterate	5.5	9.1	1.5	0.025
	Diploma	77.2	74.4	83.1	
	Academic education	17.3	16.5	15.4	
Mother’s education (%)	Illiterate	6.3	11.0	2.1	0.006
	Diploma	82.1	73.6	83.6	
	Academic education	11.7	15.3	14.4	

^a^One-way ANOVA and chi-squared analyses were used

*mMED* modified Mediterranean diet

**Table 2:** Dietary intake of study participants among tertiles of mMED diet score

**Components of mMED diet score**	**Tertiles of mMED diet score**			*P*-value^a^
	T1	T2	T3	
Fruits (g/1000 kcal)	75.66 ± 61.37	86.92 ± 73.34	107.90 ± 78.54	< 0.001
Vegetables (g/1000 kcal)	79.88 ± 54.83	91.04 ± 71.05	104.04 ± 60.36	< 0.001
Whole grain (g/1000 kcal)	190.74 ± 73.54	192.19 ± 73.92	196.29 ± 62.49	0.671
Legumes (g/1000 kcal)	27.79 ± 21.87	33.10 ± 27.92	37.14 ± 23.35	< 0.001
Nuts (g/1000 kcal)	4.85 ± 9.84	6.11 ± 8.87	7.14 ± 7.07	0.012
Fish (g/1000 kcal)	2.46 ± 3.79	3.59 ± 7.11	3.68 ± 3.44	0.016
MUFA/SFA	15.70 ± 6.17	12.41 ± 5.44	10.67 ± 4.29	< 0.001
Total dairy (g/1000 kcal)	180.28 ± 99.71	143.14 ± 90.57	113.40 ± 75.88	< 0.001
Total Red meat (g/1000 kcal)	7.85 ± 6.53	6.44 ± 5.71	6.20 ± 5.44	0.003
**Dietary nutrient intakes**				
Total energy (Kcal)	2404.47 ± 764.67	2872.64 ± 803.78	3157.31 ± 731.18	< 0.001
Protein (%)	13.74 ± 2.28	13.50 ± 2.21	13.56 ± 2.13	0.435
Carbohydrate (%)	53.61 ± 7.47	55.37 ± 7.40	56.65 ± 6.43	< 0.001
Total fat (%)	34.67 ± 8.10	33.60 ± 7.62	32.46 ± 6.72	0.004
Cholesterol (g/1000 kcal)	115.69 ± 58.77	86.56 ± 50.56	73.62 ± 42.22	< 0.001
SFA (g/1000 kcal)	14.68 ± 5.45	10.80 ± 4.60	8.45 ± 3.47	< 0.001
MUFA (g/1000 kcal)	15.70 ± 6.17	12.41 ± 5.44	10.67 ± 4.29	< 0.001
PUFA (g/1000 kcal)	10.30 ± 4.86	8.50 ± 4.30	7.70 ± 3.53	< 0.001
Dietary fiber (g/1000 kcal)	15.89 ± 5.78	17.07 ± 6.59	18.01 ± 5.27	< 0.001
Sodium (mg/1000 kcal)	1911.02 ± 843.30	1617.28 ± 763.77	1405.63 ± 458.00	< 0.001
Potassium (mg/1000 kcal)	1358.28 ± 263.59	1377.81 ± 304.44	1428.25 ± 242.34	0.012
Vitamin A (mcg/1000 kcal)	230.80 ± 316.11	208.97 ± 114.36	224.49 ± 123.66	0.627
Beta-carotene (mcg/1000 kcal)	1156.55 ± 876.37	1187.83 ± 820.35	1508.84 ± 1080.82	< 0.001
Vitamin C (mg/1000 kcal)	32.45 ± 18.94	35.68 ± 23.43	41.61 ± 19.62	< 0.001
Vitamin E (mcg/1000 kcal)	4.95 ± 2.20	4.98 ± 1.95	5.26 ± 1.81	0.198
Iron (mg/1000 kcal)	7.06 ± 1.45	7.55 ± 1.49	7.83 ± 1.33	< 0.001
Folate (mcg/1000 kcal)	223.36 ± 50.05	228.87 ± 49.98	237.11 ± 45.83	0.006
Calcium (mg/1000 kcal)	459.66 ± 141.62	408.94 ± 123.18	378.35 ± 112.02	< 0.001

Data reported as mean ± SD

^a^Obtained from One-way ANOVA

*mMED* Modified Mediterranean diet

The association between the mMED diet score and insomnia level are presented in Table 3. There was an inverse association between the mMED diet score and insomnia level in the crude model (β = -0.091, 95% confidence interval (CI): -0.392 to -0.046); *P*-value = 0.013). As well, this relationship remained after adjustment for confounding factors in Model I (β = -0.098, CI: -0.423 to -0.045; *P* = 0.015), Model II (β = -0.092, CI: -0.410 to -0.029; *P*-value = 0.024), and Model III (β = -0.082, CI: -0.385 to -0.006; *P* = 0.044).

**Table 3:** The association between mMED diet score and insomnia score

	Beta	*P*-value	95% Confidence Interval for Beta	
			Lower Bound	Upper Bound
Crude	-0.091	0.013	-0.392	-0.046
Model I	-0.098	0.015	-0.423	-0.045
Model II	-0.092	0.024	-0.410	-0.029
Model III	-0.082	0.044	-0.385	-0.006

Model I, Adjusted for energy intake; Model II, Additionally adjusted for mensturation status, physical activity, father’s education, and mother’s education; Model III, Additionally adjusted for age, BMI percentile, and abdominal obesity

*mMED* Modified Mediterranean diet, *BMI* body mass index

## Discussion

The present study revealed an inverse association between the mMED diet score with insomnia level among adolescent girls. The result of our study is in line with some previous studies. Rosi et al. [19] reported that adherence to Mediterranean diet was directly related to sleep duration in adolescents and sleep duration was sufficient in the medium and high adherence to Mediterranean diet categories. A cross-sectional study by Adelantado-Renau et al. [18] demonstrated that the effect Mediterranean diet on academic performance was mediated by sleep quality in Spanish adolescents. Godos et al. [29] found that high adherence to the Mediterranean diet is associated with better sleep quality just among normal or overweight adults but not in obese individuals. Moreover, in a cross-sectional study on Arab women was revealed that women with higher adherence to the Mediterranean diet had significantly better sleep and lower insomnia symptoms [30].

The Mediterranean diet contains a high content of fruits, vegetables, whole grains, nuts, seafood, and olive oil. It has been demonstrated that bioactive components of these food items such as antioxidants and anti-inflammatory substances can reduce oxidative stress and act as a neuroprotective agent [31]. During sleep deprivation, the rate of oxidative processes in several organs like the brain, heart, and liver increases, and this neuro-inflammation directly contributed to poor sleep [32, 33]. In addition, the richness of the Mediterranean diet in plant foods and seeds contains different levels of melatonin, and serotonin can be associated with sleep–wake brain centers [34, 35]. Evidence has shown that melatonin has a key role in sleep initiation and low levels of serotonin can cause sleep disorders [36, 37]. The Mediterranean diet contains seafood, nuts, and seeds with high content of tryptophan known as an amino acid related to the regulation of the circadian rhythm. It is demonstrated that tryptophan is the most beneficial promotor of sleep [38].

Some fruits and vegetables’ biomarkers (B-carotene and lycopene) are positively associated with sleep duration [39]. Moreover, it is demonstrated that some vitamins, including vitamin C and D have a direct link with sleep duration and quality [40]. A study by Jansen et al. [41] on young adults reported that more consumption of fruit and vegetables is related to better sleep features (quality and onset latency) in men. Another study found that a low intake of vegetables and fruits associated with a short sleep duration and poor sleep quality [42]. It has been shown that low quality diet, which is usually characterized by high consumption of sugar-sweetened beverages, processed and fast foods, and a lower intake of fresh fruits and vegetables is significantly associated with low sleep duration among students aged 6–18 years old [43]. Although red meat is an important source of high quality protein that can be used as precursors of melatonin, a previous study reported that higher red meat intake may lead to poor sleep quality [44].

Further studies have established a bidirectional link between inflammatory biomarkers, such as C-reactive protein (CRP) and interleukin-6 (IL-6 and), and sleep disorders [40, 45]. The high content of phytochemicals and PUFA in the Mediterranean diet can affect inflammatory biomarkers through some neuroprotective activities including, anti amyloidogenic efficacy and neural mediators modulation [46–48]. Albeit, we should consider that the intake of olive oil and seafood, as important sources of PUFA and MUFA, is very low in our country and these food items could not be available to many people. On the other hand, the high amount of MUFA and PUFA intake is related to the high consumption of vegetable oils among individuals.

There are some strength points for our study. High quality of data collection by validated questionaires was first strength point in the current study. In addition, to avoid misleading conclusions in analysis and interpretation of data, we conducted rigorous statistical analyses, including several adjustment models for confounding factors to depression. Nevertheless, our findings require to be interpreted by considering some potential limitations. Cross-sectional design should be considered as the major limitation of the present study; because, we do not confirm a causal relationship. FFQs are prone to measurement error and misclassification. Also, the current study was performed only on girls and not boys that this might be a limitation. Finally, like other observational studies, several unmeasured confounders were in this study, which we are unable to control them.

## Conclusion

Our study demonstrated an inverse relationship between the mMED diet score and insomnia levels among Iranian adolescent girls. The Mediterranean diet can improve sleep quality because it is a source of many food groups containing melatonin and other effective compounds. Further studies, especially randomized clinical trials, are required to clarify the effect of the Mediterranean diet on insomnia.

## Data Availability

The data and materials of the current study is available from the corresponding author on reasonable request.
